# Electroacupuncture in conscious free-moving mice reduces pain by ameliorating peripheral and central nociceptive mechanisms

**DOI:** 10.1038/srep34493

**Published:** 2016-09-30

**Authors:** Ying Wang, Jianxun Lei, Mihir Gupta, Fei Peng, Sarah Lam, Ritu Jha, Ellis Raduenz, Al J. Beitz, Kalpna Gupta

**Affiliations:** 1 Vascular Biology Center, Division of Hematology, Oncology & Transplantation, Department of Medicine, University of Minnesota, 14-100 PWB, 516 Delaware ST SE, Minneapolis, MN 55455, USA; 2 Department of Neurosurgery, University of California San Diego, 200 West Arbor Drive #8893, La Jolla, CA 92103-8893, USA; 3 Department of Veterinary and Biomedical Sciences, University of Minnesota, 295 Animal Science/Veterinary Medicine Bldg, 1988 Fitch Ave, St. Paul, MN 55108, USA

## Abstract

Integrative approaches such as electroacupuncture, devoid of drug effects are gaining prominence for treating pain. Understanding the mechanisms of electroacupuncture induced analgesia would benefit chronic pain conditions such as sickle cell disease (SCD), for which patients may require opioid analgesics throughout life. Mouse models are instructive in developing a mechanistic understanding of pain, but the anesthesia/restraint required to administer electroacupuncture may alter the underlying mechanisms. To overcome these limitations, we developed a method to perform electroacupuncture in conscious, freely moving, unrestrained mice. Using this technique we demonstrate a significant analgesic effect in transgenic mouse models of SCD and cancer as well as complete Freund’s adjuvant-induced pain. We demonstrate a comprehensive antinociceptive effect on mechanical, cold and deep tissue hyperalagesia in both genders. Interestingly, individual mice showed a variable response to electroacupuncture, categorized into high-, moderate-, and non-responders. Mechanistically, electroacupuncture significantly ameliorated inflammatory and nociceptive mediators both peripherally and centrally in sickle mice correlative to the antinociceptive response. Application of sub-optimal doses of morphine in electroacupuncture-treated moderate-responders produced equivalent antinociception as obtained in high-responders. Electroacupuncture in conscious freely moving mice offers an effective approach to develop a mechanism-based understanding of analgesia devoid of the influence of anesthetics or restraints.

Chronic pain remains a major unmet therapeutic challenge in large part because opioids, the mainstay of pain treatment, are associated with side effects such as addiction, tolerance, hyperalgesia and end-organ damage^1^. Refractory pain and adverse side effect profiles of opioid medications have led to increased use of integrative and alternative analgesic modalities such as acupuncture^2^,^3^. Acupuncture has been extensively investigated for different chronic pain conditions^4^. The majority of studies into the mechanisms underlying acupuncture have been conducted using restrained or anesthetized animals^5^,^6^,^7^,^8^. Mouse models are powerful platforms to examine pain-related mechanisms and outcomes in different pathological conditions, but the small size and often aggressive nature of some mouse strains pose challenges to performing interventions in conscious animals. However, the clinical administration of acupuncture analgesia is principally reliant on the achievement of “Qi,”^9^ representing a central phenomenon of awareness and consciousness^10^. Some studies used a sub-optimal dose of anesthetics in rat models of electroacupuncture to reduce the effect of anesthetics^11^,^12^. Anesthetics are known to influence nociception and may confound studies of pain pathobiology^13^,^14^. It is thus critical to study methods such as acupuncture in conscious, unrestrained animals. We sought to address these challenges by developing methods to perform acupuncture in conscious, freely moving mice without anesthesia or restraint.

Because of its superior effect^15^, electroacupuncture is used more frequently than manual acupuncture for animal models with pain conditions^6^,^8^,^16^,^17^,^18^. However, the analgesic response to electroacupuncture varies among individual patients as well as among individual animal models of pain^19^,^20^,^21^. In this regard gene expression profiles from the central nervous system of restrained rats following electroacupuncture showed changes in neuroimmune modulators and accompanying antinociceptive response^19^,^20^. Electroacupuncture also ameliorated moderate cerebral ischemia in rats^22^.

With regard to sickle cell disease (SCD), inflammation and ischemia play critical roles in the pathobiology of this disease^23^. We have shown that inflammation, neuroinflammation and neurogenic inflammation contribute to chronic hyperalgesia in sickle mice^24^,^25^,^26^,^27^,^28^. Because SCD is a genetic disease, pain can start in infancy and require lifelong opioid analgesic therapy. Effective long-term pain therapies must not only treat sickle pain, but also have minimal side effects that do not diminish quality of life. Emerging data demonstrate the analgesic effectiveness of electroacupuncture/acupuncture in patients with SCD^29^.

We utilized transgenic HbSS-BERK sickle mice and controls expressing normal human hemoglobin A on the same genetic background (HbAA-BERK) of both genders to establish a technique for electroacupuncture in freely moving, conscious mice. This mouse model recapitulates the hematologic effects, end-organ pathology and pain observed clinically^24^,^30^,^31^,^32^,^33^,^34^. For validation, we included an additional transgenic mouse model showing the evolutionary spectrum of breast cancer starting from ductal atypia, leading to tumor progression, metastasis, hyperalgesia and reduced survival^35^,^36^. Since pain driven by underlying chronic pathobiology in transgenic models is different than chemically evoked models, we also included the well studied complete Freund’s adjuvant (CFA)-evoked hyperalgesia model. Using a comprehensive array of mechanical, thermal and deep-tissue hyperalgesic behavioral pain assays, we examined the anti-nociceptive effect of electroacupuncture and associated peripheral and central mechanisms in freely moving, conscious mice.

## Results

### Electroacupuncture produces persistent antinociception in conscious, free-moving mice for mechanical hyperalgesia

**Figure 1:**
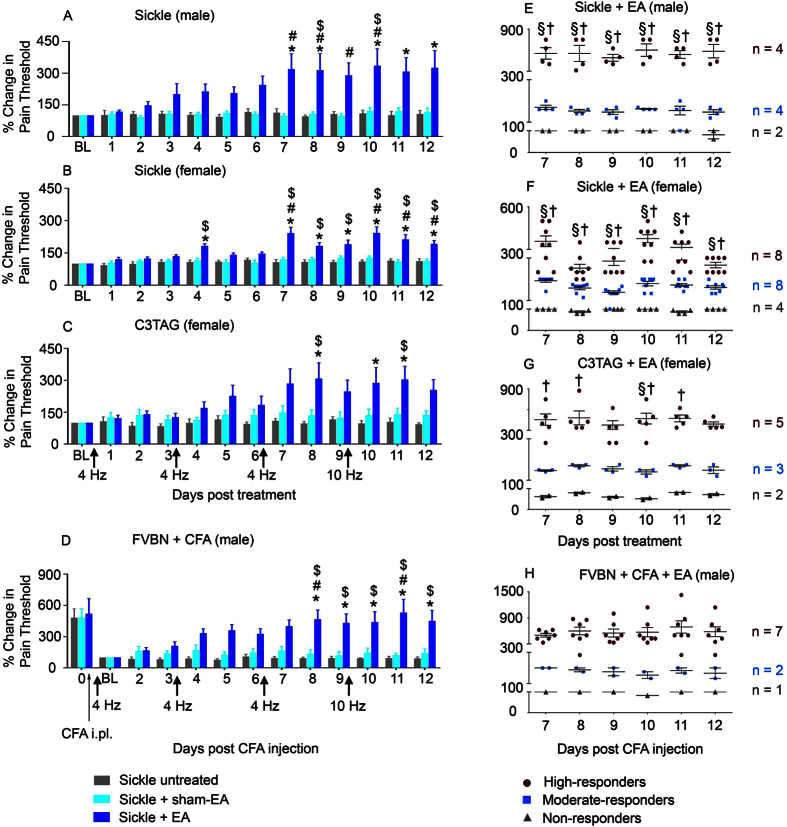
Electroacupuncture results in varied antinociceptive responses in three separate pain models. Mechanical hypealgesia in (**A**) male (n = 8–10/group) and (**B**) female sickle mice (n = 18–20 per group), (**C**) female transgenic C3TAg mice with painful tumors (n = 8–10 per group), and (**D**) male FVB/N mice with complete Freund’ adjuvant (CFA)–induced inflammatory pain (n = 8–10 per group) were treated with four consecutive electroacupuncture (EA, frequency: 4–10 Hz, intensity: #3 out of 10, pulse width: 100 μm, duration: 30 min) at day 0 (baseline: BL) and days 3, 6, and 9. Pain measures were obtained before starting electroacupuncture treatments on day 0 (baseline, BL), and daily until day 12 before each electroacupuncture treatment. Similar pain measures were also performed on sham treated (Sham-EA) or untreated mice (Untreated). Paw withdrawal frequency (PWF) threshold response to mechanical allodynia was determined (see Methods) and expressed as the % change in pain threshold. (**E–H**) Based on the percent change in pain threshold, mice were grouped as high- (nociceptive threshold increase >200%), moderate (nociceptive threshold increase 100~200%) and non-responders (nociceptive threshold increase ≤100%) for days 7–12. *p < 0.05 vs BL of matching group; ^$^p < 0.05 vs untreated; ^#^p < 0.05 vs sham-EA (two-way ANOVA, Tukey); ^§^p < 0.05 vs EA moderate-responders; ^†^p < 0.05 vs EA non-responders. Data are mean ± SEM of values normalized to BL for (**A–C)** and normalized to day 1 post-CFA injection and electroacupuncture treatment for (**D)**. Abbreviations: BL: baseline; complete Freund’s adjuvant: CFA; EA, electroacupuncture; i.pl.: intraplantar; PWF: paw withdrawal frequency.

We first tested the efficacy of electroacupuncture in awake/conscious and freely moving mice without anesthesia in 3 different mouse models: [i] transgenic homozygous BERK sickle mice with chronic pain, [ii] transgenic C3TAG mice with spontaneous breast cancer, showing the evolutionary spectrum of human breast cancer, and [iii] FVBN mice with CFA-induced inflammatory pain. Both genders of sickle mice show characteristic features of chronic pain observed clinically, including mechanical, thermal, and deep tissue hyperalgesia^24^,^31^,^33^,^37^. On the basis of preliminary experiments, we selected optimal treatment parameters for electroacupuncture administration (frequency: 4 Hz, pulse width: 100 μs, duration: 30 min). Three 30-minute treatment sessions at a frequency of 4 Hz and a final 30-minute treatment session at 10 Hz were applied at 3-day intervals over 9 days. For male sickle mice, a gradual increase in paw withdrawal threshold (PWT) to mechanical stimuli started on day 3 after the first electroacupuncture treatment and reached significance on day 7 (p < 0.05 vs baseline (BL), and sham-electroacupuncture (sham-EA) treated mice; Fig. 1A). This analgesic effect was sustained through day 12, which was 3 days after the final electroacupuncture treatment on day 9 and represented the final observation period in our experimental paradigm. In female sickle mice hyperalgesia was significantly attenuated on day 4 and from days 7 through 12 (p < 0.05 vs BL and untreated mice; Fig. 1B). However, it was not significantly different on days 5 and 6, perhaps due to variability in the estrous cycle of female mice. We did not observe a statistically significant difference in hyperalgesia at different stages of the estrous cycle (Supplementary Fig. 1A,B), but on day 6 some of the mice were in estrous (Supplementary Fig. 1C). While it is likely that the stage of the estrous cycle would influence the level of analgesia in response to acupuncture treatment, as evidenced by our finding that there are fluctuating analgesic responses across the estrous cycle in untreated female mice, this was not a focus of the present study and we did not pursue this avenue in the present study. With that in mind electroacupuncture was uniformly started at the pre-estrous stage of each female mouse to decrease the influence of the stage of the estrous cycle. Based on the data in Fig. 1, it is also likely that female sickle mice require a more robust treatment regimen or higher frequency of stimulation to obtain comparable sustained analgesia to male mice. In this regard, electroacupuncture was relatively more effective on the day immediately after the treatment in female mice (days 7 and 10 after electroacupuncture treatments on days 6 and 9, respectively), while in male sickle mice the effect persisted and was progressively greater with successive treatments.

Next we examined whether identical electroacupuncture treatment produced anti-nociception in the mouse model of breast cancer and in a CFA-induced inflammatory pain model in FVB/N mice. Electroacupuncture significantly increased the pain threshold on day 8 in both female C3TAG mice (p < 0.05 vs BL and untreated mice, Fig. 1C) and male mice with CFA-induced inflammatory pain (p < 0.05 vs BL, untreated and sham-EA treated mice; Fig. 1D). Similar to the observations on female sickle mice, C3TAG female mice showed significantly improved anti-nociception after electroacupuncture treatment, which decreased over time. It is likely that female mice may require more frequent electroacupuncture treatments. On the other hand, in both male sickle and CFA-induced male FVBN mice, electroacupuncture resulted in a sustained anti-nociceptive effect.

We observed varied antinociceptive responses among identical electroacupuncture-treated mice within each mouse model. To analyze individualized responses, we categorized all treated mice into three groups on the basis of their increase in pain threshold after electroacupuncture treatment: *high-responders* (>200%), *moderate*-*responders* (100~200%), and *non-responders* (≤100%) (Fig. 1E–H). The percent change in pain threshold was calculated from data collected during days 7 to 12 because of the consistent anti-noceceptive effect during this period in different groups of mice. Approximately 40–50% of transgenic sickle mice of both genders and C3TAG mice were high responders. Additionally, about 30–40% of both genders of sickle mice and C3TAG mice were moderate responders, and about 20% were non-responders to electroacupuncture. In contrast, 70% of CFA-induced FVBN male mice were high responders, and only 20% and 10% were moderate or non-responders, respectively. These general differences in electroacupuncture response between transgenic sickle or C3TAG mice in comparison to CFA-induced mice suggests that in chronic disease states the variability in the pathobiology may account for increased variability in response to electroacupuncture treatment. Although acute treatment with CFA leads to more uniform nociception resulting in more uniform response to electroacupuncture, it is likely that variable organ damage and injury in sickle mice and C3TAG mice affect different locations of the body, which may be more responsive to different acupoints. In addition, it is also possible, though unlikely, that some variability in electroacupuncture response may occur in electroacupuncture administration to relatively small-sized mice.

Overall, these data demonstrate that electroacupuncture can be effectively administered to awake, free-moving mice leading to significant analgesia in transgenic mice with chronic nociception due to disease pathology as well as in a model of acute inflammatory pain.

### Electroacupuncture attenuates characteristic features of cold or musculoskeletal hyperalgesia in male and female sickle mice

**Figure 2:**
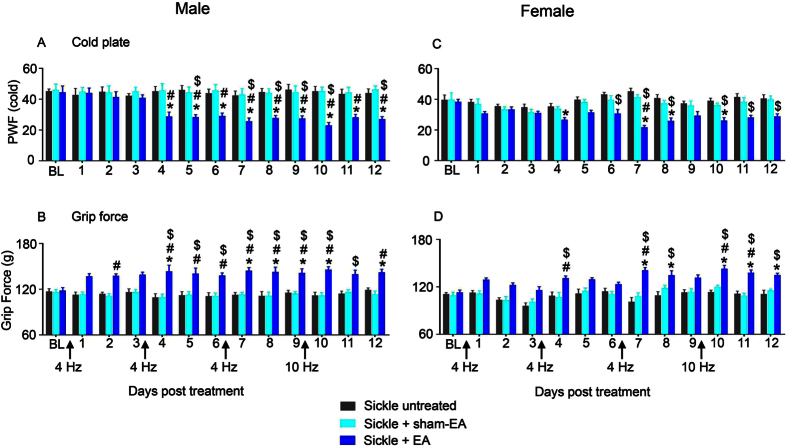
Electroacupuncture attenuates chronic hyperalgesia in sickle mice. Male (**A,B**) and female (**C,D**) sickle mice (HbSS) were treated with four consecutive electroacupuncture (EA) treatments as described in Fig. 1. Measures of (**A,C**) thermal sensitivity to cold (PWF), and (**B,D**) deep tissue pain (grip force) are shown. *p < 0.05 vs BL of matching group; ^$^p < 0.05 vs Sickle untreated; ^#^p < 0.05 vs Sickle + sham-EA (two-way ANOVA, Tukey). Each value is the mean ± SEM from 8–10 mice with 3 observations per mouse. Abbreviations: BL: baseline; EA, electroacupuncture; PWF, paw withdrawal frequency.

In male sickle mice, electroacupuncture decreased paw withdrawal frequency (PWF) to a cold stimulus and increased grip force as a measure of deep/musculoskeletal hyperalgesia in a sustained manner from day 4 onwards (Fig. 2A,B). In female sickle mice, electroacupuncture resulted in a significant and sustained anti-nociceptive effect after 7 days of electroacupuncture treatment for cold and deep/musculoskeletal hyperalgesia (Fig. 2C,D). Thus, male sickle mice were more responsive to electroacupuncture than the female sickle mice. Electroacupuncture did not have any effect on the hyperalgesic response of HbAA control mice throughout the entire experimental period (Supplementary Fig. 2).

### Electroacupuncture attenuates SCD associated systemic inflammation

Acupuncture has been suggested to ameliorate inflammation and nociception by influencing inflammatory cytokines/chemokines^38^. Consistent with previous studies, we observed increased levels of white blood cells (WBCs) and reticulocytes, and decreased hematocrit in whole blood of sickle mice as compared to control mice (Supplementary Table 1). All levels of responders to electroacupuncture treatment had comparable hematocrit and reticulocytes in whole blood. However, WBCs were significantly decreased in high responders (p < 0.05 vs HbSS untreated), but not in moderate or non-responders. Importantly, non-responders had significantly higher WBC counts than high- or moderate-responders (p < 0.05 vs Sickle + EA high-responders), suggesting greater systemic inflammation.

**Figure 3:**
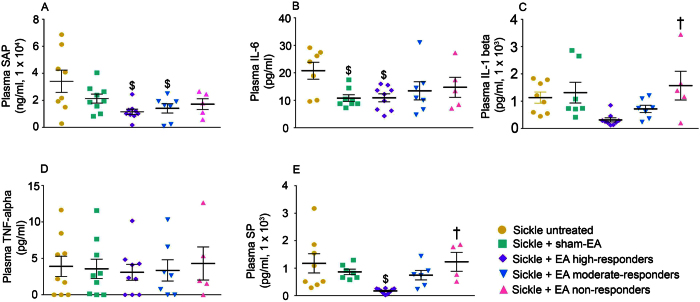
Electroacupuncture reduces systemic inflammation in sickle mice. Levels of (**A**) serum amyloid P (SAP), (**B**) IL-6, (**C**) IL-1beta, (**D**) TNF-alpha, and (**E**) substance P (SP) in plasma of HbSS-BERK sickle mice were measured on day 13 after the last nociceptive assessment. Electroacupuncture (EA)-treated sickle mice were grouped in high- (n = 9–10), moderate- (n = 6–7), and non-responders (n = 4–5) according to the varied antinociceptive responses observed in Fig. 1. Levels in untreated (n = 7–9) or sham treated (n = 7–9) sickle mice were were also assesed. ^$^p < 0.05 vs Sickle untreated; ^†^p < 0.05 vs Sickle + EA high-responders (one-way ANOVA, Tukey). Data are presented as mean ± SEM. Abbreviations: EA, electroacupuncture; SAP, serum amyloid P; SP: substance P.

To address the mechanisms that are potentially responsible for electroacupuncture’s various antinociceptive effects and hematological patterns, we sought to define the potential mediators involved in suppressing both inflammation and hyperalgesia in high- and moderate-responders to electroacupuncture treatment. In SCD, systemic levels of several inflammatory cytokines and substance P (SP) are elevated in the blood of sickle mice, and in patients with SCD^25^,^28^,^39^. We found that serum amyloid P (SAP), a marker of general inflammation^40^, was decreased 2-fold in high- and moderate-responders after electroacupuncture treatment, suggesting reduced inflammation (Fig. 3A; p < 0.05 vs untreated sickle mice). Similarly, IL-6 and IL-1beta were significantly or largely reduced in the plasma of high responders (Fig. 3B,C; p < 0.05 vs untreated mice), but not in moderate or non- responders. TNF-alpha was unaffected by electroacupuncture treatment (Fig. 3D). Surprisingly, IL-1beta levels were significantly higher in low responders as compared to untreated sickle mice (Fig. 3C). Moreover, sham electroacupuncture treatment led to a significant decrease in IL-6 levels (p < 0.05 vs untreated mice). Plasma SP levels were significantly reduced by electroacupuncture treatment in high responders (Fig. 3E, p < 0.05 vs untreated mice), but not in moderate or non-responders. These data show that the systemic inflammatory milieu, including cytokines and SP, are significantly reduced in high responders to electroacupuncture, suggesting that electroacupuncture influences the inflammatory microenvironment in sickle mice.

### Influence of electroacupuncture on peripheral inflammation and mast cell activation

**Figure 4:**
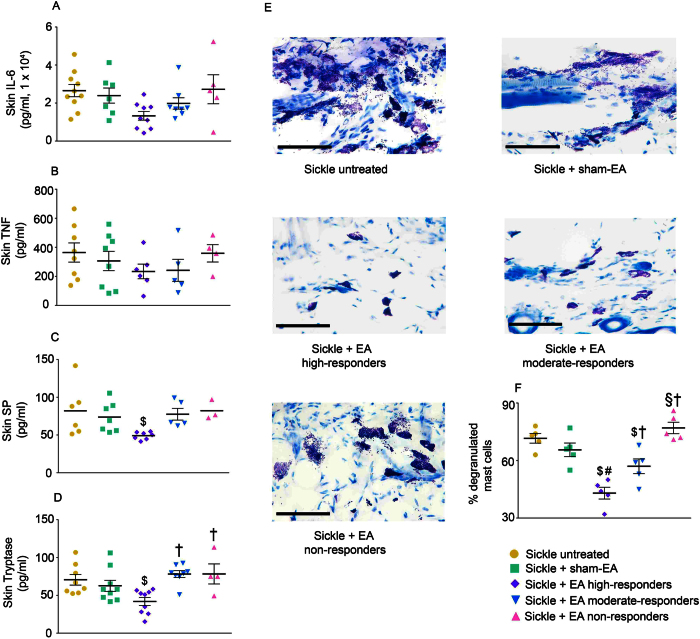
Electroacupuncture reduces peripheral inflammation and mast cell activation in sickle mice. Levels of (**A**) IL-1beta, (**B**) substance P (SP), (**C**) IL-6, and (**D**) TNF-alpha from cultured skin biopsies were compared between untreated (n = 6–11) or sham treated (n = 7–9) sickle mice, as well as electroacupuncture (EA)-treated high- (n = 6–9), moderate- (n = 5–8) and non-responder (n = 3–5) sickle mice. (**E**) Representative images of toluidine blue-stained mast cells in skin for untreated or sham treated sickle mice, as well as electroacupuncture-treated high-, moderate-, and non-responder sickle mice (n = 5 for each group). Scale bar, 10 μm. (**F**) Degranulated mast cells as percentage of total mast cells (n = 5 for each group). ^$^p < 0.05 vs Sickle untreated; ^#^p < 0.05 vs Sickle + sham-EA; ^†^p < 0.05 vs Sickle + EA high-responders (one-way ANOVA, Tukey for D and F, Dunnett’s for C). Data are presented as mean ± SEM. Abbreviations: EA, electroacupuncture; SP: substance P.

We have previously shown that mast cells are activated in the skin of sickle mice and contribute to neurogenic inflammation by releasing SP^25^. To assess the interactions between mast cells and nerve fibers, we examined the release of inflammatory cytokines and SP in the the dorsal skin of mice. There was a trend towards a reduction in IL-6 levels in high responders, but TNF-alpha levels were unaffected by electroacupuncture treatment (Fig. 4A,B). In contrast, there was a significant decrease in the release of both SP and tryptase from the skin in high responders (Fig. 4C,D; p < 0.05). Complementary to decreased tryptase levels, mast cell activation was significantly reduced in both high responders and moderate responders (Fig. 4E,F; p < 0.05). Together, these data suggest that mast cell activation and associated nerve fiber stimulation likely contribute to SP release, which is modulated by electroacupuncture.

### Electroacupuncture ameliorates neurogenic inflammation

**Figure 5:**
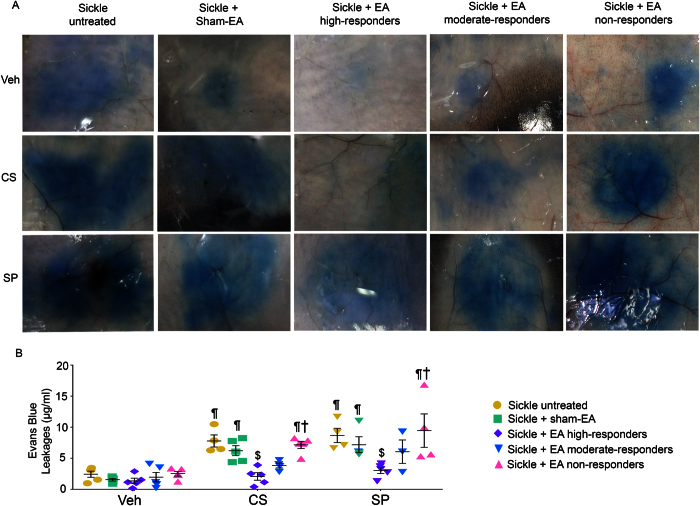
Electroacupuncture ameliorates neurogenic inflammation evoked by substance P or capsaicin in sickle mice. (**A,B**) Evans blue leakage evoked by substance P (SP), capsaicin (CS), or vehicle (Veh) in untreated or sham treated sickle mice, as well as electroacupuncture (EA)-treated high-, moderate-, and non-responder sickle mice. Each image represents reproducible images from 5 mice each, and each value is the mean ± SEM from 5 male mice. ^$^p < 0.05 vs Sickle untreated; ^†^p < 0.05 vs Sickle + EA high-responders; ^¶^p < 0.05 vs vehicle of matching group (one-way ANOVA, Tukey). Abbreviations: EA, electroacupuncture; CS, capsaicin; SP: substance P; Veh, vehicle.

Neuropeptides, including SP and calcitonin gene related peptide (CGRP), released from peripheral nerve fibers induce venular permeability and arteriolar dilatation, leading to neurogenic inflammation. This can in turn enhance the excitabilty of nociceptors and the release of inflammatory cytokines such as IL-1beta^41^. Given that electroacupuncture reduced the levels of SP in plasma and skin tissue, we speculated that it would likely ameliorate neurogenic inflammation, which is an outcome of peripheral nerve activation. To test this hypothesis, microvascular permeability was quantified by Evans blue leakage after intradermal injection of capsaicin or SP (Fig. 5A). SP and capsaicin administration produced significantly greater vascular leakage than vehicle. In addition, this increased leakage of Evans blue evoked by intradermal administration of SP or capsaicin was significantly inhibited by electroacupuncture treatment in high-responders, but not moderate- or non-responders, as compared with untreated mice (Fig. 5B; p < 0.05). These findings suggest that electroacupuncture’s antinociceptive effect in SCD may in part be due to its effect on neurogenic inflammation.

### Electroacupuncture attenuates central nociceptive mechanisms

**Figure 6:**
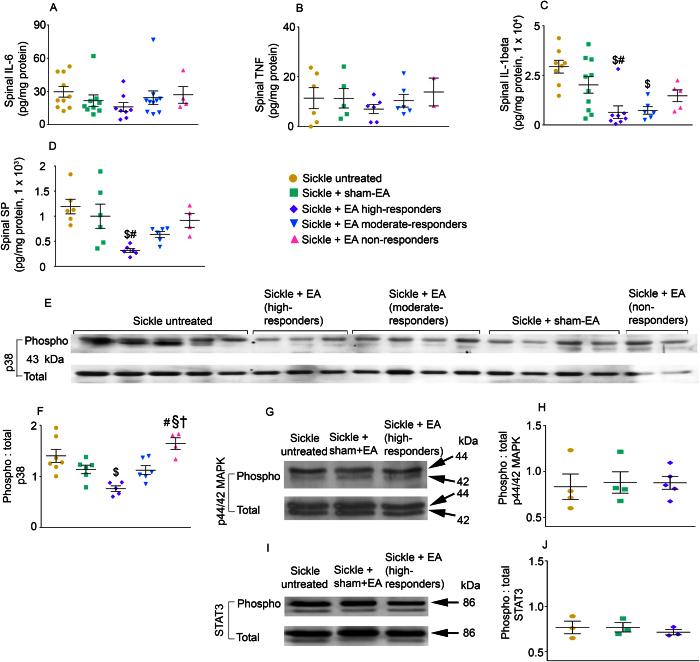
Electroacupuncture attenuates spinal cord nociception in sickle mice. Levels of (**A**) IL-6, (**B**) TNF-alpha, (**C**) IL-1beta, and (**D**) SP were measured in spinal cord lysates of HbSS-BERK sickle mice collected after the last day of nociceptive assessment. Electroacupuncture-treated sickle mice were grouped as high- (n = 6–8), moderate- (n = 6–10), or non-responders (n = 2–5). Analysis of untreated (n = 6–10) or sham treated (n = 5–10) sickle mice is also shown. (**E,F**) Phosphorylation level of p38 MAPK relative to total p38 MAPK in the spinal cord of untreated (n = 7), sham treated (n = 6), and EA-treated high- (n = 5), moderate- (n = 6), or non-responder (n = 4) sickle mice. (**G–J**) Phosphorylation level of p44/42 MAPK or STAT3 relative to total p44/42 MAPK or STAT3 in the spinal cord of untreated, sham treated, or EA-treated high-responder sickle mice (n = 3–5 in each group). ^$^p < 0.05 vs Sickle untreated; ^#^p < 0.05 vs Sickle + sham-EA; ^§^p < 0.05 vs Sickle + EA moderate-responders; ^†^p < 0.05 vs Sickle + EA high-responders (one-way ANOVA, Tukey). Data are presented as the mean ± SEM. Abbreviations: EA, electroacupuncture; SP: substance P.

In sickle mice, spinal cord inflammatory cytokines IL-6 and TNF-α remained unresponsive to electroacupuncture, but IL-1beta showed a significant decrease in both high- and moderate-responders (Fig. 6A–C; p < 0.05). In high responders, electroacupuncture also led to a significant decrease in spinal SP and phosphorylation of p38 MAPK, two critical mediators of chronic pain (Fig. 6D–F; p < 0.05). In contrast, spinal MAPK/ERK and STAT3 phosphorylation remained unaltered following electroacupuncture treatment (Fig. 6G–J; p > 0.05). These data suggest that electroacupuncture attenuates key central nociceptive mechanisms of chronic pain by reducing SP and p38 MAPK phosphorylation.

### Electroacupuncture reduces the requirement of analgesic opioids in moderate responders

**Figure 7:**
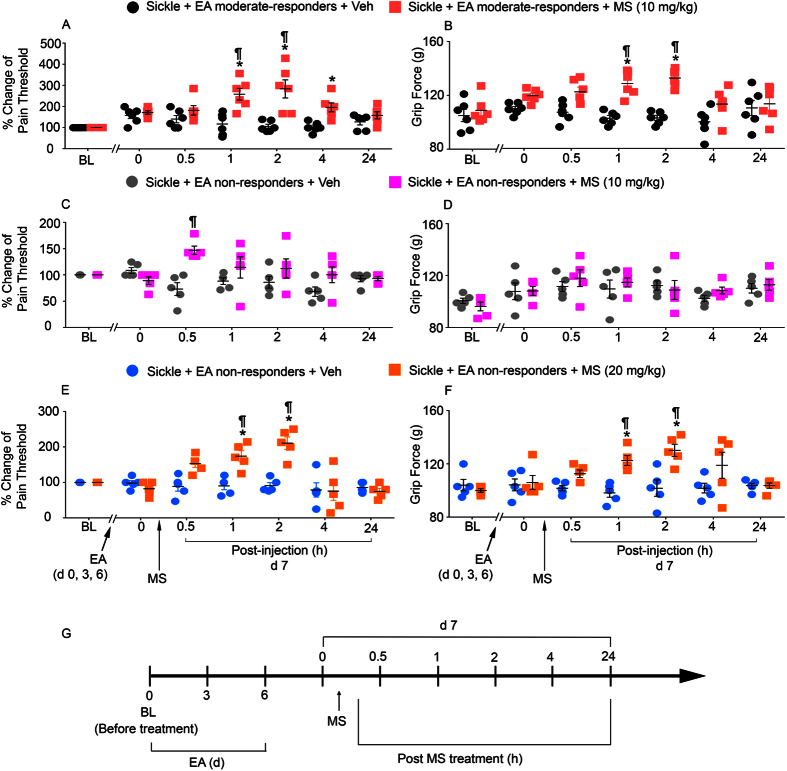
Morphine enhances electroacupuncture-induced attenuation of chronic hyperalgesia in sickle mice. HbSS-BERK sickle mice were treated with three consecutive electroacupuncture treatments as described in Fig. 1, and grouped according to their nociceptive response as in Fig. 1. At day 7, morphine (MS, 10 mg/kg) or vehicle (Veh) solution was administered to electroacupuncture-treated (**A,B**) moderate- (n = 6) or (**C,D**) non-responder (n = 5) sickle mice. (**E,F**) Non-responders also received 20 mg/kg morphine (n = 5). (**A,C,E**) PWF threshold response to mechanical allodynia is expressed as % change of pain threshold, and (**B,D,F**) deep tissue pain is measured by grip force. (**G**) Measurements were taken at BL (before electroacupuncture treatment) and day 7; on day 7 multiple measurements were taken: time 0 (before MS/Veh administration) and 0.5, 1, 2, 4, and 24 h after MS/Veh injection. *p < 0.05 vs BL of matching group; ^¶^p < 0.05 vs Veh at same time point (two-way ANOVA, Tukey). Data are presented as the mean ± SEM. Abbreviations: BL: baseline; EA, electroacupuncture; MS, morphine; Veh, vehicle.

High-dose opioids are required to treat sickle pain in mice and humans^24^,^32^. In previous studies, we found that 20 mg/kg morphine is required to ameliorate hyperalgesia and that 10 mg/kg morphine did not have an analgesic effect in sickle mice^24^,^25^. However, we observed that moderate responders showed significant, time-dependent improvement in pain threshold and grip force after treatment with a sub-optimal dose of morphine (10 mg/kg) in combination with electroacupuncture (Fig. 7A,B; p < 0.05). In non-responders, we observed an increase in pain threshold, but not in grip force, after treatment with a combination of 10 mg/kg morphine and electroacupuncture (Fig. 7C,D, p < 0.05). Furthermore, we observed a sustained increase in both pain threshold and grip force in non-responders after treating the mice with 20 mg/kg morphine in combination with electroacupuncture (Fig. 7E,F; p  < 0.05). These novel data demonstrate that a sub-optimal dose of morphine when combined with electroacupuncture can reduce hyperalgesia in moderate sickle mice responders, but not in non-responders. However, high-dose morphine remains effective in those who do not respond to electroacupuncture.

## Discussion

In the present study we have developed and established a novel method for electroacupuncture delivery that provides a sustained anti-nociceptive effect in conscious, free-moving mice without restraint or anesthesia in a variety of different models of pain. Our procedure represents a significant advancement over previously used protocols, in which electroacupuncture-induced pain control has been examined in mice exposed to the confounding influences of anesthetics or restraint^5^,^8^. Thus, our method of electroacupuncture administration offers the advantage of investigating the mechanisms of electroacupuncture-induced pain control without the confounding influences of anesthetics or restraint-induced stress, which is accompanied by a complex pattern of hormonal responses and functional changes in the central nervous system. Since electroacupuncture is typically performed on awake/conscious and unrestrained patients, our procedure in mice simulates the procedures performed in human subjects. Moreover, we used transgenic mouse models that represent the disease progression seen in human breast cancer and SCD, as well as the constitutive hyperalgesia that humans with these diseases experience. In contrast, mechanisms of acupuncture analgesia have been examined in a wide variety of animal models where experimentally induced pain was evoked by chemical irritants^6^,^42^, cell implantation^43^, nerve ligation^44^,^45^, incision^46^,^47^, or surgery^48^,^49^. Comparisons between chronic pain in transgenic models with that of CFA-induced inflammatory pain, show the difference in response to electroacupuncture, thus suggesting the use of mouse models of chronic disease to clearly understand the translational potential of pre-clinical findings.

In the present study, we also found that sham treatment inconsistently elicited comparable effects as electroacupuncture in both genders, although sham treatment-induced antinociception was discontinuous and statistically insignificant compared with verum electroacupuncture. This finding is consistent with clinical evidence from large randomized controlled trials indicating that verum acupuncture produces specific and superior antinociceptive effects in comparison to sham acupuncture^50^,^51^.

Classical studies show the involvement of opioid peptides in EA analgesia^52^,^53^. As described by Han 2003, low (2–4 Hz) and high frequency (100–200 Hz) EA analgesia is mediated by different opioid peptides, namely β-endorphin and dynorphin, respectively^53^. In earlier studies the effect of EA at 4 Hz, 1 ms, 25 T intensity (=25 times threshold) was blocked by intrathecally as well as intravenously administered naltrexone prior to EA, but not when administered after the EA, thus arguing for a preventive effect^54^. Therefore, it is likely that the analgesic effect observed with EA using 4 Hz in our study is due to the release of β-endorphin.

It is noteworthy that less than 50% of sickle mice of both genders and 50% of cancer mice were high responders as compared to 70% of mice with CFA-induced inflammatory pain. There has been very few studies in the literature that have characterized individuals into high, moderate and nonresponders to acupuncture treatment. The fact that a certain percentage of animals and human patients do not respond to acupuncture treatment is important for acupuncturists to know. Our results are among the first to provide a mechanistic explanation for these differences with respect to variations in cytokines and substance P. This variability in the pathobiology of our transgenic models of pain is consistent with the heterogeneity seen clinically in SCD and cancer patients^34^. Importantly, female sickle mice were relatively less responsive to electroacupuncture analgesia as compared to male sickle mice, who showed sustained analgesia over a longer period of time. Consistent with this finding analgesic therapy in hospitalized SCD patients has been reported to be extended in female as compared to male patients, which might be attributed to the more chronic and refractory neuropathic pain in female patients^55^. This gender-specific variability in response does not appear to be related to the estrous cycle in females, and may therefore be due to gender-specific pathobiologic and/or nociceptive mechanisms. Indeed, recent studies demonstrate differences in mechanisms of nociception in male and female mice^56^. Thus, using mouse models that represent the natural clinical condition of both genders, devoid of anesthesia, may provide a relatively improved translational outcome with increased relevance for defining treatable targets.

Despite the fact that increasing evidence supports the potential efficacious effects of acupuncture in reducing cancer-associated pain^57^,^58^ and arthritis/osteoarthritis associated musculoskeletal pain in patients^59^,^60^, only one clinical report has been published demonstrating significantly reduced pain in acupuncture-treated SCD patients for chronic or acute pain^29^. SCD patients that do use complementary and alternative medicine to reduce pain primarily rely on prayer, relaxation techniques, and massage rather than acupuncture^61^. Our results suggest that acupuncture may also be considered in treating SCD patients.

Because of gender-specific responses to analgesic therapies in both humans and animals^62^, we included male and female sickle mice in our study in order to evaluate the efficacy of acupuncture in both genders. Increasing evidence supports an augmented pain sensitivity in late proestrous and estrus stages of the estrous cycle compared to the metestrous and diestrous stages^63^,^64^,^65^,^66^,^67^,^68^,^69^. In line with these findings, we observed fluctuating analgesic responses across the estrous cycle in untreated female mice. Interestingly, electroacupuncture appeared to interfere with estrous-linked cyclic pain sensitivity, because reduced von Frey-evoked mechanical sensitivity was only observed on the day after verum electroacupuncture treatment, which was not seen in untreated or sham-treated mice. Pursuant to the principles of traditional Chinese medicine, acupuncture treatment is not recommended during menstruation unless it is for regulating the menses, although the scientific evidence for this principle remains unclear. This contradiction was difficult to address in the chronic treatment protocol used in our studies. This may partly account for the less profound and unsustainable analgesia found in female mice compared to their male counterparts. In this regard, we found different patterns of individual responses in female and male sickle mice, where most female high-responders showed modestly increased pain thresholds (between 200~400%) in comparison to the high thresholds found in male sickle mice (300~700%). Similarly, we previously found that female sickle mice exhibit greater mechanical and thermal hyperalgesia than males^24^. This is consistent with reports showing that female subjects exhibit higher pain sensitivity and varied analgesic responses than males in a variety of pain conditions and in response to opioid treatment^62^,^70^. Conversely, electroacupuncture-treated male sickle mice exhibited sustained analgesic response to cold and deep tissue hyperalgesia from day 7 until the last period of observation, although electroacupuncture-induced attenuation of mechanical allodynia was not as significant as cold/deep tissue hyperalgesia at individual time points. Gender differences in cold pain threshold have also been reported among healthy volunteers receiving acupuncture treatment^71^.

More importantly, we found varying antinociception in electroacupuncture-treated mice in all three models examined in the present study. To date, evaluation of individual response differences for acupuncture analgesia studies is sparse, although it is easy to surmise that the reports in both human and animal subjects with acupuncture treatment exhibit varied outcomes. One earlier study characterized rats based on tail flick latency into high- and low-responders^72^. The mechanisms remain unknown for individuals who barely respond to acupuncture or who do not respond at all. A cDNA array analysis of patients treated with acupuncture suggested that the varied responses to acupuncture analgesia among individuals may be more determined by inherited factors than by psychological factors^73^. Such individual differences were previously mentioned with respect to acupuncture analgesia in a rat model of inflammatory pain^18^. Acupuncture-induced variable analgesia in animals may be also due to more uncontrolled factors such as limited acupoint distribution and manipulations due to the small size of mice.

To better elucidate the variable response to electroacupuncture in SCD, we analyzed the modulation of both peripheral and central mechanisms in groups of mice showing high, moderate, and low responses to electroacupuncture in comparison to sham and untreated mice. Pain in SCD is accompanied by general inflammation, neuro-inflammation, and mast cell activation^74^,^75^. Both peripheral and central nociceptive neuroinflammatory processes have been demonstrated in sickle mice by us and others^25^,^26^,^76^,^77^. In sickle mouse models, there is a dramatic increase in SAP, a mouse homologue to human serum C-reactive protein, as well as the proinflammatory cytokines IL-6, TNF-a, and IL-1beta^40^,^78^,^79^. Electroacupuncture significantly suppresses the proinflammatory cytokines TNF-alpha and IL-1beta and inflammatory pain in peripheral inflamed tissue induced by CFA^42^ or carrageenan^80^. Our data showing that electroacupuncture produces a significant decrease in the levels of SAP in high and moderate responders, but not in non-responders, is consistent with a correlation between the levels of inflammatory mediators and levels of nociception. In addition, SP and IL-1beta were reduced in high-responders, whereas all responders along with sham-treated mice displayed lower levels of IL-6 compared to untreated controls. Electroacupuncture-induced decrease in the levels of IL-6 and SP were also observed in the secretagogue from skin biopsies, an indicator of peripheral modulation. The concomitant decrease in mast cell activation and its marker tryptase in high responders may further contribute to the reduced release of cytokines and SP. Additionally, neurogenic inflammation in response to the release of SP from nerve fibers was also attenuated in high responders, suggestive of a neuromodulatory effect of electroacupuncture in the periphery.

Chronic hyperalgesia in sickle mice appears to evoke both peripheral and central sensitization^76^,^77^,^81^,^82^. Additionally, elevation in SP is observed in both sickle mice^25^ and patients^31^,^39^. Our results showing that electroacupuncture significantly reduces SP and SCD-induced nociception would suggest an association between SP levels and nociception in sickle mice. Electroacupuncture has also been shown to significantly reduce the expression of SP and its receptor in the colon in rats with irritable bowel syndrome^83^ and in the dorsal horn of the spinal cord in S180 mice with bone cancer^84^. In agreement with previous studies, we observed that electroacupuncture reduced the levels of SP and IL-1beta in the periphery, as well as in the spinal cord in high-responders (Fig. 6C,D). In addition to SP, spinal p38 MAPK signaling is also critical in the processing of chronic pain^85^. In previous studies, we observed increased phosphorylation of p38 MAPK in association with constitutive sensitization of second order neurons in the spinal cord of sickle mice^82^. In the present study, we also observed a significant decrease in spinal levels of phosphorylated p38 MAPK in high-responders, but not in moderate or non-responders. Electroacupuncture did not influence p44/42 MAPK/ERK or STAT3 signaling. Collectively these studies suggest that electroacupuncture attenuates the inflammatory and neuroinflammatory milieu in the periphery as well as in the central nervous system. As p38 MAPK, SP and IL-1beta are critical mediators of chronic pain, their persistent presence in moderate and non-responders may be contributing to the poorer outcomes observed in these mice.

We recently demonstrated that spinal glial cell activation occurs in SCD, which accompanies the increased levels of SP^26^. It has previously been reported that electroacupuncture reduces the activation of microglia and astrocytes as well as levels of IL-1beta and SP in the spinal cord of rats with neuropathic pain^86^. Electroacupuncture also significantly suppresses spinal microglial activation, SP, and IL-1beta in a monoarthritis pain model induced by CFA^87^. Moreover, disruption of glial function synergizes with the electroacupuncture-induced analgesic response^87^,^88^. Therefore, it is likely that electroacupuncture’s ability to inhibit chronic hyperalgesia in SCD may be associated with decreased activation of spinal glia and astrocytes, which would therefore reduce the release of spinal nociceptive neuromodulators. The increased release of inflammatory neuropeptides (e.g., CGRP and SP) is also thought to contribute to the abnormal vasculature and axonal sprouting found in sickle mice^24^. In a spinal cord injury model, electroacupuncture treatment was shown to increase the expression of 5-Hydroxy tryptamine-positive nerve fibers and axons, as well as levels of neurotropin-3 and glial fibrillary acidic protein (GFAP) in injured spinal cord tissue^89^. In the periphery we have shown that electroacupuncture can alter nerve fibers and vascular and lymphatic distribution in peripheral tumors; these changes are associated with a reduction in tumor-induced nociception^43^. Collectively these data raise the possibility that electroacupuncture may modify nerve fiber and vascular regeneration both peripherally and centrally in SCD via the modulation of specific mediators (e.g., SP), leading to improved vascularity and decreased pain.

Together, our data suggest that amelioration of multiple peripheral and central nociceptive signatures is required to attain analgesia in SCD. It is likely that variable responses to electroacupuncture in different patients may be an outcome of heterogeneous neurochemical and inflammatory modulation in the periphery and central nervous system. Once recognized, inhibition of relevant mediators in combination with other strategies may improve analgesia with electroacupuncture in moderate or non-responders.

In the present study, we achieved similar analgesia with a sub-optimal dose of morphine (10 mg/kg) in combination with electroacupuncture in moderate-responders as compared to our previous finding with a high dose (20 mg/kg) of morphine in sickle mice without any treatment^24^. Sickle mice show an analgesic response to 20 mg/kg morphine, but not to 10 mg/kg^24^. Our observations on reduced requirement of morphine in moderate responders are in agreement with the reduced requirement of opioid analgesia post-operatively for abdominal surgery, following pre-operative acupuncture treatment at low and high frequency^90^. Since, opioids are required in extremely high doses and for long periods of time to treat sickle pain, strategies such as acupuncture may reduce the requirement of opioids to achieve analgesia and thereby reduce their side effects.

Our data demonstrate the effectiveness of electroacupuncture to ameliorate hyperalgesia and abrogate peripheral and central mediators of inflammation and nociception in sickle mice. It is noteworthy that electroacupuncture’s therapeutic and mechanistic effects, however, were variable, with a minority of sickle mice not responding to electroacupuncture treatment, and it is likely that SCD patients may show similar variability in response to electroacupuncture. However, collectively these observations on electroacupuncture in conscious free moving mice, support the use of electroacupuncture for analgesia in SCD and therefore appropriate clinical trials to evaluate the efficacy of electroacupuncture are warranted. Hydroxyurea therapy in SCD has led to reduced mortality in patients with SCD, but it does not prevent or reduce SCD-associated pain according to the evidence-based guidelines for SCD^91^. It is plausible that electroacupuncture may improve overall outcomes of SCD when combined with hydroxyurea therapy. Thus, acupuncture may be a beneficial adjuvant approach to the current limited pain management therapies in SCD.

## Methods

### Animals

All procedures described were approved by University of Minnesota’s Institutional Animal Care and Use Committee and were conducted in accordance with the statutes of the Animal Welfare Act and the guidelines of the Public Health Service as issued in the Guide for the Care and Use of Laboratory Animals (revised 2011). Three different mouse models were used in this study. First, to model sickle cell-induced nociception, we used a transgenic HbSS-BERK (called sickle mice) expressing >99% human sickle hemoglobin and littermate HbAA-BERK (called control mice) expressing normal human hemoglobin A^25^. Mice between 5–7 months of age were phenotyped for the presence of human sickle hemoglobin S (HbS) and normal human hemoglobin A by isoelectric focusing as described^27^ and genotyped by Transnetyx services (Cordova, TN). Second, to model breast cancer pain, we used 5–7 month old female transgenic mice carrying the C3TAG (C3(1)/simian virus 40 large tumor antigen) fusion gene that develop highly invasive breast tumors associated with increased nociception. Third, to model inflammatory pain, we used male FVB/N mice aged 5–7 month where inflammatory pain in the left paw was induced by intraplantar injection of CFA (Sigma-Aldrich; 10 μg in 10 μl)^92^.

### Electroacupuncture treatment

Mice were habituated to handling in the experimenter’s hands at least three days before the initial treatment. Handling of mice requires more caution due to their aggressive behavior. Both sickle and control mice were randomly divided into electroacupuncture (EA), sham-electroacupuncture (sham-EA), and untreated groups (n = 8–10/group). The acupoint GB30 was selected in these experiments because this point is one of the most commonly used acupoints in animal models producing significant antinociception^16^,^93^. Before insertion of needles, the fur above acupoint GB30 was shaved on the lower back and disinfected. In the human body, GB30 is located at a point that is at the junction of the lateral 1/3 and medial 2/3 of the line connecting the prominence of the greater trochanter and the last sacral vertebrae, the analogue point for rat or mouse was previously descried by other investigators^16^,^94^ including us^18^,^42^. Disposable acupuncture needles (Ø = 0.16 mm, length = 13 mm, Lhasa OMS, Weymouth, MA) were inserted bilaterally into GB30. The length of the needle was shortened to 9 mm before use. The dimension of the needles was selected based on preliminary experiments and was based on the size of the mice used in this study and the thickness of the skin and underlying tissue at the GB30 acupoint. The duration of stimulation lasted for 30 min/session, and 4 sessions (frequency: 4–10 Hz, pulse width: 100 μs) were applied.

We selected low frequency of 4 Hz because it is more effective in reducing inflammatory pain and activates mu opioid receptors^95^,^96^. Moreover, In earlier studies the effect of EA at 4 Hz, was blocked by intrathecally as well intravenously administered naltrexone prior to EA, but not when administered after the EA^54^. As described by Han, low (2–4 Hz) and high frequency (100–200 Hz) EA analgesia is mediated by different opioid peptides, namely β-endorphin and dynorphin, respectively^53^. We have found that mu opioid receptors are under-expressed in sickle mice^24^. Therefore, by applying 4 Hz we could achieve higher activation of mu opioid receptors and also the release of β-endorphin to promote analgesia.

The transcutaneous electrical stimulus apparatus (Maxtens 1000, Isokinetics, Inc., Grannis, AR) connected to the needles that was used in EA for applying 4–10 Hz frequency is similar to that used in a previous study^43^. Direct irritation of nerves or blood vessels was avoided. As shown in Supplementary Video 1 of an EA treatment, the mouse exhibited a slight twitching of the lower limbs while the electrical current was gradually increased. Mice were observed carefully and did not exhibit any signs of stress or discomfort during the electroacupuncture procedure. Based on our preliminary experiments, the #3 of the 10 intensity settings on the apparatus was found to be tolerable for the majority of mice. The #3 intensity was measured and characterized using an oscilloscope (B&K Precision Model 2120), and an output current of 1.7 mA was calculated with a resistance of 10 kilo Ohm. This resistance that was measured represents the sum of all wires, wire connections, needles and the mouse itself. The voltage at the #8 intensity setting on the transcutaneous electrical stimulus apparatus was also measured as a reference (Supplementary Table 2). Therefore, depending on the type of apparatus used, this intensity scale may require minor adjustment to duplicate the exact current parameters applied in this study. A tolerable intensity of stimulation was used for each individual mouse, which allowed for conscious, free-moving behavior during the electroacupuncture treatment. However, it should be noted that there were subtle differences in the tolerable intensity of electrical stimulation between each mouse, which might contribute to individual variations. An identical protocol was used for sham-electroacupuncture treatment except that electrical stimulation was not performed.

### Drug treatment

Morphine sulfate in saline was subcutaneously administered at 10 or 20 mg/kg on day 7 in a group of EA-treated HbSS-BERK female mice.

### Nociceptive measurements

All behavioral tests were conducted in a quiet room maintained at a constant temperature of 23–25 °C by an examiner blinded to the treatment groups. Mice were allowed to acclimate to the testing room for a minimum of 60 min prior to each set of measurements. All mice were familiarized with each testing paradigm by performing a set of repeated measurements before collecting baseline measurements. Evaluation of pain behaviors were performed 24 h after the first electroacupuncture treatment and daily thereafter, prior to subsequent treatments. We included an exclusion criteria based on mechanical hyperalgeia such that only sickle mice that displayed a paw withdrawal frequency (PWF) response to 7 out of 10 applications (70%) and only non-hyperalgesic control mice that displayed a PWF of less than 3 (30%) were selected for further studies.

#### Mechanical hyperalgeia (von Frey)

PWF and paw withdrawal threshold (PWT) were obtained by von Frey monofilaments (Stoetling Co.) as described previously^25^,^97^. Briefly, PWF was determined by the frequency of paw withdrawal in response to 10 trials of application of a 1.4 g monofilament with a calibrated bending force of 12.8 mN to random points on the plantar surface of the mouse hindpaw. PWT was obtained by application of a series of monofilaments ranging from 0.07–2.0 g. Ascending or descending stimuli were applied in a consecutive fashion starting with the 1.0 g monofilament (filament 5) in the middle of the series. The testing sequence progressed following an up-down sequence such that a positive response to a particular filament indicated the next lower value filament be used in the subsequent test and a negative response indicated the next higher value filament be used. Testing was stopped if a positive response to the lowest possible filament or a negative response to the highest possible filament was observed. Withdrawal, biting, or licking was regarded as a positive response to von Frey application. Withdrawal thresholds to von Frey filaments were determined when animals lifted the hind paw at least 5 times out of 10 applications. The minimum force of the von Frey filament that evoked at least 5/10 responses was considered as the mechanical withdrawal threshold. Changes in nociceptive thresholds are presented as % change (values/baseline * 100).

#### Grip force and cold hyperaldesia

Deep tissue hyperalgesia (forepaw grip force) was measured using a wire mesh attached to a computerized grip force meter. Cold hyperalgesia (PWF) was determined on a cooled aluminum plate at 4 °C (UGO Basile Model 35100) as described previously^24^.

### Measurement of estrous cycle

Estrous stage was assessed in a vaginal secretion smear as previously described^98^ every 4 days for female sickle mice.

### Hematological analysis

Blood was collected by cardiac puncture. Hematocrits were measured as the percentage volume of red cells in heparinized glass capillary tubes after centrifugation at 500 g for 10 minutes. WBCs were counted on a hemocytometer after the RBCs were immediately lysed with 2% acetic acid containing 30 μg/ml EDTA, 4 replicates per mouse. Reticulocytes were determined microscopically in 4 random areas on each blood smear slide stained with Wright-Giemsa solution^25^,^40^.

### Quantification of cytokines/neuropeptides by ELISA

Blood, organs, and tissues were collected after the last nociceptive testing.

#### Plasma

Whole blood was collected by cardiac puncture and transferred to EDTA-tubes. Plasma was then isolated within 30 min by centrifugation for 7.5 minutes at 2500 rpm at 4 °C.

#### Spinal cord

Spinal cords were homogenized and lysed with a chilled buffer (1 M HEPES, 5 M NaCl, 1 M MgCl_2_, 0.5 M EDTA and 10% Triton X-100) cocktail containing protease inhibitors (100 mM vanadate, 1 M β-glycerophosphate, 1 mg/ml leupeptin and 50 mM a-PMSF). The supernatant was collected after centrifugation (12,000 rpm, 10 min, 4 °C). Protein levels were quantified by the Bradford method using a commercial protein assay kit (Bio-Rad).

#### Skin culture biopsies

Dorsal skin punch biopsies (4 mm diameter) were obtained, and washed in DMEM containing 10,000 units/mL penicillin G sodium, 10,000 units/mL streptomycin sulfate, and 2.5 mg/mL amphotericin B. Biopsies were then incubated overnight in DMEM plus antibiotics with 2 mM L-glutamine and 10 mM HEPES at 37 °C/5% CO_2_. The culture medium was subsequently collected for the analysis for cytokines and neuropeptides.

Isolated plasma, spinal cord lysate, and skin culture conditioned medium were analysed with commercial ELISA kits for IL-6, TNF-alpha, and serum amyloid P (all from R&D Systems), substance P (Abcam), IL-1beta (PeproTech), and tryptase (BlueGene).

### Western blot analysis of signaling pathways

Spinal cord lysates (30 μg protein) were resolved by electrophoresis and immunoblotted with antibodies to phospho-p38 MAPK (9211S), phospho-p44/42 MAPK (9101S), phospho-STAT3 (9145S), as well as unphosphorylated total p38 MAPK (8690L), p44/42 MAPK (9102L) and STAT3 (4904) (all from Cell Signaling Technology) as described previously^24^.

### Mast cell staining with toluidine blue

Skin biopsies were embedded in Tissue-Tek^®^ O.C.T. compound (Sakura Finetek (Torrance, CA)), and skin sections (6 μm) were cut and subsequently incubated at room temperature for 20 s in a toluidine blue working solution (pH: 2.3–2.5) containing 10% toluidine blue stock solution (Sigma-Aldrich, 1% dissolved in 70% alcohol) and 90% sodium chloride (1% dissolved in distilled water, pH 2.3) as described by us previously^25^. Mast cells were counted using an IX70 inverted microscope (Olympus Corporation) in 10 randomly selected fields (600× magnification). The ratio of degranulated mast cells to total mast cells was calculated as described earlier^25^.

### Quantification of plasma extravasation by Evans blue

Neurogenic inflammation was evoked by intradermal injection of SP and capsaicin on the dorsal skin, and plasma extravasation was detected by the Miles assay as previously described^25^. Briefly, the dorsal skin of the lower back was shaved 24 h before the experiment, and mice were anesthetized using 2% isoflurane prior to Evans blue injection. Evans blue dye (50 mg/kg) was administered intravenously into the tail vein 5 min before injection of 50 μL of SP (100 nM), capsaicin (1.6%), or vehicle (10% ethanol, 7.5% Tween 80 in saline) into 4 sites of exposed dorsal skin. These sites were randomly selected with at least 20 mm distance between each site. Mice were subsequently euthanized 30 min postinjection. Digital images of the back were collected using a digital camera (Samsung ST150F) with a resolution of 16 MP. The amount of dye was analyzed after collecting and weighing the dorsal skin and incubating it with formamide at 56 °C for 24 h to release Evans blue. The released Evans blue was quantified spectrophotometrically at 620 nm.

### Statistical Analysis

All data were analyzed using Prism software (v6.0e, GraphPad Prism Inc., San Diego, CA). One-way and two-way analysis of variance (ANOVA) with Tukey’s post hoc test were used for comparisons within a group and between groups, respectively. A p-value of <0.05 is considered significant. All data are presented as mean ± SEM.

## Additional Information

**How to cite this article**: Wang, Y. *et al*. Electroacupuncture in conscious free-moving mice reduces pain by ameliorating peripheral and central nociceptive mechanisms. *Sci. Rep.* **6**, 34493; doi: 10.1038/srep34493 (2016).

## Supplementary Material

Supplementary Information

Supplementary Information
