# Supplementary material for: Electroacupuncture in conscious free-moving mice reduces pain by ameliorating peripheral and central nociceptive mechanisms
Source: Sci Rep. 2016 Sep 30;6:34493. doi: 10.1038/srep34493 (PMC5043286; doi:10.1038/srep34493)
Supplement: Supplementary Information [file srep34493-s2.pdf]

Title: Electroacupuncture in conscious free-moving mice reduces pain by ameliorating peripheral and central nociceptive mechanisms.

Ying Wang[1*], Jianxun Lei[1], Mihir Gupta[2], Fei Peng[1], Sarah Lam[1], Ritu Jha[1], Ellis Raduenz[1], Al Beitz[3] and Kalpna Gupta[1*]

[1]Vascular Biology Center, Division of Hematology, Oncology & Transplantation, Department of Medicine, University of Minnesota, Minneapolis, MN, [2]Department of Neurosurgery, University of California San Diego, La Jolla, CA, [3]Department of Veterinary and Biomedical Sciences, University of Minnesota, Minneapolis, MN, USA.

Address correspondence to:

*Kalpna Gupta, Ph.D., Room 14-100, Philip-Wangensteen Building, 516 Delaware Street SE, Minneapolis, MN 55455, USA. Phone: 612.625.9604; E-mail: gupta014@umn.edu.

*Ying Wang, M.D., Ph.D., Room 14-100, 516 Delaware Street SE, Minneapolis, MN 55455, USA. Phone: 612.624.7104; E-mail: wang5729@umn.edu.

Key words: Pain, hyperalgesia, sickle cell disease, cancer, acupuncture.

Supplementary video S1: Electroacupuncture is accomplished in awake, free-moving mice. The landmarks (the bilateral great trochanter and last four sacral vertebras were marked after the fur on lower back was shaved) for positioning the GB30 acupoint were labeled before treatment was applied to the mouse. Disposable acupuncture needles were held with alcohol-sanitized hands. The needle was swiftly inserted through the skin into the subcutaneous tissue associated with the acupoint GB30. The electrodes were connected with bilateral needles and the electricity was delivered by an electrical stimulator. Sham treatment of electroacupuncture was identically performed except the electrical current was not delivered. During the treatment, the mouse did not exhibit discomfort or stress and was allowed free mobility inside the cage.

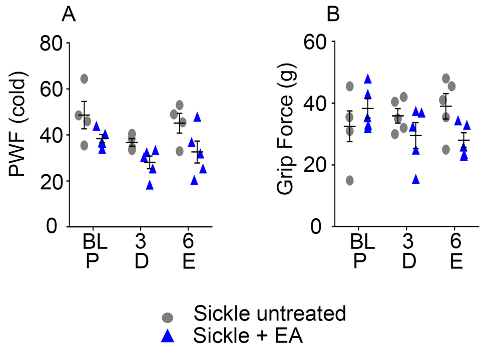

C

Days / Cages	BL	Day 3	Day 6
Sickle + EA	P	D	E
Sickle untreated	P	D	E

P: pre-estrus, E: estrus, D: diestrus

Supplementary Figure S1: Anti-hyperalgesia of electroacupuncture was insignificantly influenced by estrus stages of female sickle mice. Measures of (**A**) thermal sensitivity to cold (paw withdrawal frequency, PWF) and (**B**) deep tissue pain (grip force) were compared only between untreated female sickle mice (Sickle untreated) and electroacupuncture (EA) treated female sickle mice (Sickle + EA) who were in the identical estrus stage. (**C**) The estrus stages were displayed at BL (baseline) and on days 3 and 6. Abbreviations: EA, electroacupuncture; BL: baseline; PWF: paw withdrawal frequency.

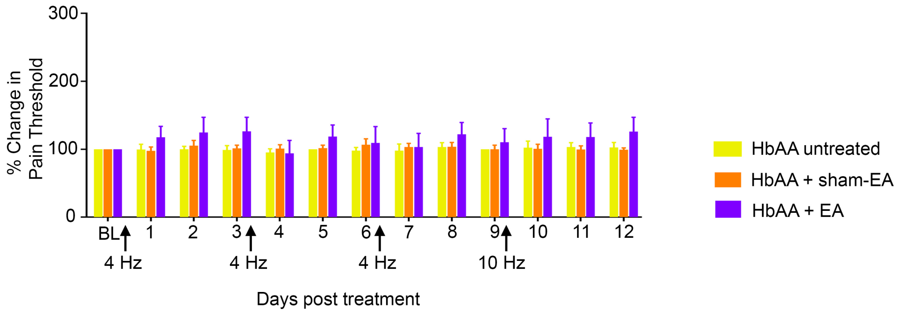

Supplementary Figure S2: **Electroacupuncture did not change the hyperalgesic level of control mice (HbAA, male).** Male sickle male were treated with four consecutive electroacupuncture (EA) treatments (frequency: 4-10 Hz, intensity: 3 mA, pulse width: 100 μm, duration: 30 min) at day 0 (baseline: BL) and days 3, 6, and 9. Pain measures were obtained before starting electroacupuncture treatments on day 0 (baseline, BL), and daily until day 12 prior to each electroacupuncture treatment (Sickle + EA, n=10) or sham treatment (Sickle + sham-EA, n=10). Similar pain measures were also performed on untreated mice (Sickle untreated, n=7). Paw withdrawal threshold responses to mechanical allodynia were determined as showed in Fig 1 (see Methods) and expressed as a % change in pain threshold. Data are presented as the mean ± SEM. Abbreviations: EA, electroacupuncture; BL: baseline.

Supplementary Table 1: Hematological parameters

Groups / Parameters	HbAA Untreated (n = 10)	HbAA + Sham-EA (n = 9)	HbAA + EA (n = 7)	Sickle Untreated (n = 10)	Sickle + sham-EA (n = 10)	Sickle + EA high-responders (n = 7-8)	Sickle + EA moderate-responders (n = 6-7)	Sickle + EA non-responders (n = 4-5)
WBCs, K/µl	6.3 ± 0.6	6.7 ± 1.1	6.2 ±0.6	26.9 ± 1.4 * ‡ £	22.0 ± 2.7 * ‡ £	11.2 ± 1.4 $ #	22.6 ± 2.7 * ‡ £ †	27.7 ± 2.2 * ‡ £ †
Hematocrit, %	49.4 ± 1.2	50.6 ± 1.2	47.5 ± 1.5	28.8 ± 2.4 * ‡ £	29.2 ± 1.7 * ‡ £	29.7 ± 2.0 * ‡ £	29.4 ± 1.6 * ‡ £	29.6 ± 1.1 * ‡ £
Reticulocyt-es, %	3.2 ± 0.3	2.9 ± 0.3	3.3 ± 0.5	32.5 ± 2.8 * ‡ £	31.6 ± 2.8 * ‡ £	30.6 ± 2.1 * ‡ £	33.3 ± 4.0 * ‡ £	32.4 ± 3.8 * ‡ £

Data present are mean ± SEM.

* p<0.05 Vs HbAA untreated;
‡ p<0.05 Vs HbAA + sham-EA;
£ p<0.05 Vs HbAA + EA;
$ p<0.05 Vs Sickle untreated;
# p<0.05 Vs Sickle + sham-EA;
† p<0.05 Vs Sickle + EA high-responders.

Abbreviations: EA, electroacupuncture.

Supplementary Table 2: Electrical intensity measurement

Intensity (#)	Resistance (ohm)	Voltage (v)	Intensity (mA)
# 3	10000	17	1.7
# 8	10000	43	4.3
